# Sketches of American Physicians—Thomas Harris, M. D.

**Published:** 1842-06-04

**Authors:** 


### *Sketches of American Physicians.*

### THOMAS HARRIS, M. D.

Dr. THOMAS HARRIS occupies a distinguished and well-earned professional position. With an extensive and lucrative general practice, he combines a high reputation as a surgeon, lecturer, and clinical instructer. He is besides one of the oldest of the medical officers in the naval service, and the "*otium cum dignitate*" which he now enjoys, is the reward of previous years of hardship and honourable exertion.

Dr. Harris was born in Chester county, in this state, on the 3d of January, 1784. He is the eldest son of the late General William Harris, who served with distinction in the war of the revolution. His paternal grandfather, a native of Ireland, was a large land-holder in the fertile valley of Chester county; his maternal grandfather, a clergyman of the Church of Scotland. The doctor received his education at the Brandywine Academy of Chester county. In the spring of 1804, he commenced the study of medicine with Dr. Davis of the same county, and, after attending the lectures at the University of Pennsylvania, obtained his degree in 1809. For three years afterwards he practised his profession in Chester county with considerable success. In 1812, during the war with Great Britain, he received from Mr. Madison a commission as surgeon in the navy, and joined the Wasp sloop of war, under the command of the gallant Commodore (then Commander) JACOB JONES. Hardly in the service, Dr. Harris had the good fortune to take part in one of the most brilliant actions of the war. A week after sailing from Newcastle, the Wasp encountered the sloop of war Frolic, of a superior force, and, after an action of little more than half an hour, captured her. An hour subsequently, however, both the prize and her captor fell into the hands of the Poictiers, seventy-four, which carried them into Bermuda. Here they remained a few weeks, until they were exchanged. Upon returning home, Captain Jones and all his officers, including of course Surgeon Harris, were ordered to the Macedonian frigate. The Macedonian was blockaded in New London for a year, and thence transferred to the lakes. After serving a year on the lakes in this ship, and in the frigate Mohawk, Dr. Harris was again ordered to the Macedonian, Captain Jones to form part of Decatur's squadron against Algiers. The Algerine frigate, Mazouda, and a brig of war, were captured by Commodore Decatur. The Mazouda was unprovided with a surgeon, and had suffered greatly during the action. Dr. Harris was placed on board of her, where he had his hands full, with amputations and other operations. After cruising along the Barbary and other ports on the Mediteranean, he returned to the United States with the squadron in the autumn of 1815.

These three years of active service gave Dr. Harris an admirable opportunity of making himself a skilful operator. He had the qualities necessary to turn his advantages to account—judgment, coolness, readiness, and dexterity—and he came out of the war with an established reputation and solid experience.

Upon returning home, Dr. Harris was placed on furlough for a year; then ordered to the Guerrière at Boston, where he remained till 1817; and afterwards stationed at the hospital of the Navy Yard at Philadelphia. At this station he has been ever since fixed, with the exception of a short cruise to the West Indies in 1823. In this year, he was sent with Commodore Rogers at the head of a commission to examine into the condition of the seamen suffering from the yellow fever at Key West, and to report as to the eligibility of that port as a station for our squadrons. During his residence in Philadelphia, Dr. Harris has been employed in various capacities in the naval service. He was chosen to select the site for the Naval Asylum in this city and to superintend its erection; and has repeatedly served on the board to examine candidates for the medical corps.

With the advantage of an excellent reputation, Dr. Harris commenced the practice of his profession in this city in 1817. His success has been brilliant. Two years ago, when he was compelled by ill health to relinquish active business, he was in the receipt of a professional income, that has seldom been reached in Philadelphia. Dr. Harris possesses in an eminent degree those minor qualifications for professional success, without which the strongest combination of talent and knowledge is unavailing. To an agreeable address, a pleasant flow of conversation, and a cordiality of manner, the more attractive because felt to be sincere, he unites a ready command of resources, therapeutic and dietetic, and the happy capacity of almost endlessly varying them, and adapting them to the tastes of his patients.

Dr. Harris has been for a number of years a lecturer on surgery. In 1823, he formed one of a private association with Drs. Hewson, Meigs, and Bache, with whom he continued till 1826, when he was appointed to lecture on Surgery in the Medical Institute. His courses in this school have been eminently popular. We have never heard a better practical lecturer. His style is familiar, sometimes conversational, and his matter has the great attraction of appearing to emanate more from his own experience than the gleanings of books. Dr. Harris has long been a champion of the non-specific doctrines of syphilis and of the anti-mercurial treatment of this disease. He devotes a considerable portion of his lectures to this subject, and defends his views ably and ingeniously. Most of our readers will probably take issue with him on this point: at least, our own opinion is, that the mass of evidence, particularly the recent experiments by inoculation, tend to confirm the view of John Hunter "that the venereal disease arises from a poison, which is capable again of producing a similar disease." Dr. Harris has had much reputation in the treatment of syphilitic affections. As he pursues a strictly anti-mercurial course, his success may fairly be adduced to show that the *primary* symptoms of the disease are very manageable without mercury. In 1826, he published an elaborate memoir on this subject in the North American Medical and Surgical Journal which was extensively copied into the European journals.

Dr. Harris was for twelve years one of the surgeons to the Pennsylvania Hospital, having held the post from 1829 to 1841, when he resigned from ill health. During this, long clinical service, he has been distinguished for the success as well as the number of his operations. In 1837, he excised the elbow-joint, for caries—the first time the operation was performed in this country. He amputated the tongue in two instances for hypertrophy. These cases were published in the American Journal for the years 1830 and 1837. A series of excellent clinical lectures by Dr. Harris have appeared in this journal.

Dr. Harris has contributed a number of articles to different medical periodicals. In 1821, he published a paper on Metastasis in the Medical Recorder, which, like the article on syphilis, went the rounds of the European journals. A life of Commodore Bainbridge, published in 1837, is extremely creditable to Dr. Harris' literary powers. This spirited sketch of the hero of the Java may fairly rank with any of our naval biographies.

For a year or two past, the state of his health has forced Dr. Harris in a measure to retire from his professional avocations. We are sincerely glad to know that his strength is so far re-established as to permit him to give his summer course of lectures. No member of the profession can claim more of the regard and respect of his brethren, and his return to active duty will give general and real gratification.

